# Supplementary material for: The Protective Effects of 2’-Fucosyllactose Against E. Coli O157 Infection Are Mediated by the Regulation of Gut Microbiota and the Inhibition of Pathogen Adhesion
Source: Nutrients. 2020 May 1;12(5):1284. doi: 10.3390/nu12051284 (PMC7282266; doi:10.3390/nu12051284)
Supplement: Supplementary file 1 [file nutrients-12-01284-s001.pdf]

nutrients

Supporting Information:

Article

The Protective Effects of 2′-Fucosyllactose against *E. coli* O157 Infection Are Mediated by the Regulation of Gut Microbiota and the Inhibition of Pathogen Adhesion

Yuanyifei Wang [1], Yan Zou [2], Jin Wang [2], Hui Ma [2], Bowei Zhang [2] and Shuo Wang [1,2,*]

1 Key Laboratory of Food Nutrition and Safety, Ministry of education, Tianjin University of Science and Technology, Tianjin 300457, China; wangyyf163@163.com
2 Tianjin Key Laboratory of Food Science and Health, School of Medicine, Nankai University, Tianjin 300071, China; 2120181361@mail.nankai.edu.cn (Y.Z.); wangjin@nankai.edu.cn (J.W.); mahui2018@mail.nankai.edu.cn (H.M.); bwzhang@nankai.edu.cn (B.Z.)
* Correspondence: wangshuo@nankai.edu.cn.; Tel.: +86-22-85358445

Received: 14 March 2020; Accepted: 23 April 2020; Published: 1 May 2020

Table S1. Primer Sequences for qPCR

gene	forward primer（5′→3′）	reverse primer（5′→3′）
Stx1	GAAGAGTCCGTGGGATTACG	AGCGATGCAGCTATTATTAA
Beta-actin	GGCTGTATTCCCCTCCATCG	CCAGTTGGTAACAATGCCATGT
TNF-α	GCTCTGTGAAGGGAATGGGTGTT	GCTCTGTGAAGGGAATGGGTGTT
IL-6	ACTTCCATCCAGTTGCCTTCTTG	AGCTGGATGCTCTCATCAGG
IL-1β	TTGACGGACCCCAAAAGAT	AGCTGGATGCTCTCATCAGG
MUC2	CTGTGCCAATGGCCTCAAAC	GCCCATCGAAGGTGACAAAG
Occludin	GGTCTCTACGTGGATCAATATTTGTA	AACCCCAGGACAATGGCTA

qPCR condition: The PCR was performed as follows: initial melting temperature at 95 °C for 60 s, 40 cycles of denaturation at 95 °C for 15 s, and 60 °C for 40 s.，draw melting curve at the end.

Table S2. Concentration of SCFAs in Feces of Each Group

group	acetic acid (μmol/g)	propionic acid (μmol/g)	butyric acid (μmol/g)	valeric acid (μmol/g)
CK	43.14 ± 8.98	12.84 ± 2.80	14.13 ± 2.64	5.09 ± 1.06
MC	43.09 ± 6.47	12.89 ± 2.88	13.91 ± 3.21	5.16 ± 1.17
FL	53.64 ± 10.80	14.77 ± 2.58	15.60 ± 2.29	5.46 ± 0.84

Data are expressed as mean ± SD (n = 8). There was no significant difference between the groups ($p > 0.05$).

© 2020 by the authors. Licensee MDPI, Basel, Switzerland. This article is an open access article distributed under the terms and conditions of the Creative Commons Attribution (CC BY) license (http://creativecommons.org/licenses/by/4.0/).
